# A First Publication by the International Labour Office

**Published:** 1921-04-16

**Authors:** 


### THE CAUSE OF INDUSTRIAL CANCER.

### A First Publication by the International Labour Office.

The International Labour Office, set up by the Peace Treaty as part of the organisation of the League of Nations and aiming at improvement of the conditions of labour in all countries by international action, has recently produced its first publication, a booklet dealing with cancer of the bladder occurring among workers in aniline factories. For the subject of Industrial Hygiene a special section has been established under Dr. Carozzi, and we trust the excellent summary which is now produced will be the forerunner of many more.

The subject of possible causes of malignant growth has a wide interest apart from its industrial application. It has long been known that tumours occur among chimney-sweeps, petrol-refiners, workers handling tar, paraffin, pitch, and in a variety of other occupations. For some of them recent work has provided a partial explanation, and a certain amount of light has thus been thrown upon one section of the cancer problem. But the idea of an internal tumour in the urinary system caused by chronic excretion of small quantities of some one substance has particular possibilities in this direction. Also, with the development of the dye industry in this country, the means of prevention forms a point of great practical interest.

### Etiological.

Following a few uncertain observations upon the condition, a long series of cases studied by Leuenberger at Basle, has apparently established a definite relation between the aniline industry and vesical cancer. There is still some doubt as to how long an exposure will produce the growth, but less than three years is claimed as sufficient in the case of benzidine, five years with naphthylamine and over ten with other substances. As might be expected, the earliest signs are simply irregular inflammation and irritation of the bladder. Later dysuria and hæmaturia develop and the patient's attention is thus drawn to the seriousness of the disease. There is apparently no immunity, and personal predisposition is marked. Among many factors favourable to this "poisoning," the most prominent are lack of cleanliness, malnutrition, alcoholism, and excesses of every kind, but workers new to the trade, and consequently working without precautions, with whom large skin surfaces come in contact with the poisonous products, are especially liable. On the other hand, those remaining a long time in the workshops are also exposed to the danger of absorbing orally floating particles from the air. Undoubtedly those people who take great care of their skins show the smallest number of poisoning cases.

### The Type of Cancer.

In type the bladder tumours are simply papillary, adenomatous, sarcomatous, or adenocarcinomatous, but the most remarkable fact about them is the extraordinary degree of polymorphism exhibited in any one instance. In an early case it is difficult to tell whether the tumour is innocent or malignant. Even microscopically different characters are shown in different parts of the same specimen. But this appearance is merely suggestive of a progressive change from simple epithelial overgrowth into typical cancerous tissue.

### A Parallel Instance.

As the *Lancet*, in dealing with the subject, has recently pointed out, there appears to be an exact parallel between this tumour production in the bladder and the causation of skin warts (and ultimately malignant growths) by various externally-applied coal-tar products. In both cases the difficulty arises in determining when the initial epithelial overgrowth has taken on malignant characters. Both conditions may quite possibly be caused by the same coal-tar constituent. In the urine this body may be automatically separated and excreted. Therefore, in the case of the vesical condition, it seems that further investigation may well result in isolation of this causal substance. In any case, it is a line well worth further research, and Kuchenbecher has already invented a colour test, making it possible to discover an animo-compound present in the urine. Later, one will hope to hear that the irritant, epithelioma-producing substance or group of substances has been definitely isolated, and therefrom the pathology of soot and coal-tar cancer will be taken one step nearer to complete elucidation. Evidence, according to the report, now points to the animo-benzenes, particularly benzidine and $\beta$-naphthylamine.

### Means of Prevention.

The practical politics of the moment call, however, for simple means to prevent absorption of the poisons. Nitration and reduction particularly are now carried out in closed, ventilated, and entirely mechanical apparatus. General cleanliness of the workshops, mechanical ventilation, and absorption of all dust are essential. Working clothes must be closed at the wrist and sleeves. A clear skin, particularly when eating, must be insisted upon, and all workers should undergo periodical medical examination, all cases of hæmaturia being compulsorily notifiable. Milk is a valuable antidote, and alcohol and smoking are best prohibited. Such measures are by no means new, and we have now several instances of their successful application in other more obvious industrial poisonings. There are, however, already signs that they are proving effective in the case of aniline absorption, and we are, therefore, on the verge, as far as the present report takes us, of an efficient prophylaxis and a valuable observational contribution to the great cancer problem.

